# A substantial fraction of phytoplankton-derived DON is resistant to degradation by a metabolically versatile, widely distributed marine bacterium

**DOI:** 10.1371/journal.pone.0171391

**Published:** 2017-02-03

**Authors:** Luca Polimene, Darren Clark, Susan Kimmance, Paul McCormack

**Affiliations:** 1 Plymouth Marine Laboratory, Prospect Place, The Hoe, Plymouth, United Kingdom; 2 Petroleum and Environmental Geochemistry Group, Biogeochemistry Research Centre, University of Plymouth, Drake Circus, Plymouth, United Kingdom; Universitat Bremen, GERMANY

## Abstract

The capacity of bacteria for degrading dissolved organic nitrogen (DON) and remineralising ammonium is of importance for marine ecosystems, as nitrogen availability frequently limits productivity. Here, we assess the capacity of a widely distributed and metabolically versatile marine bacterium to degrade phytoplankton-derived dissolved organic carbon (DOC) and nitrogen. To achieve this, we lysed exponentially growing diatoms and used the derived dissolved organic matter (DOM) to support an axenic culture of *Alteromonas* sp.. Bacterial biomass (as particulate carbon and nitrogen) was monitored for 70 days while growth dynamics (cell count), DOM (DOC, DON) and dissolved nutrient concentrations were monitored for up to 208 days. Bacterial biomass increased rapidly within the first 7 days prior to a period of growth/death cycles potentially linked to rapid nutrient recycling. We found that ≈75% of the initial DOC and ≈35% of the initial DON were consumed by bacteria within 40 and 4 days respectively, leaving a significant fraction of DOM resilient to degradation by this bacterial species. The different rates and extents to which DOC and DON were accessed resulted in changes in DOM stoichiometry and the iterative relationship between DOM quality and bacterial growth over time influenced bacterial cell C:N molar ratio. C:N values increased to 10 during the growth phase before decreasing to values of ≈5, indicating a change from relative N-limitation/C-sufficiency to relative C-limitation/N-sufficiency. Consequently, despite its reported metabolic versatility, we demonstrate that *Alteromonas* sp. was unable to access all phytoplankton derived DOM and that a bacterial community is likely to be required. By making the relatively simple assumption that an experimentally derived fraction of DOM remains resilient to bacterial degradation, these experimental results were corroborated by numerical simulations using a previously published model describing the interaction between DOM and bacteria in marine systems, thus supporting our hypothesis.

## Introduction

The marine reservoir of dissolved organic matter (DOM) is one of the largest carbon stores on Earth. The release of organic molecules from micro-organisms to seawater, either during active growth or cell death, represents a major source of this complex and chemically diverse pool to the marine environment. When freshly released, this pool represents a rich source of carbon, nutrients and energy and is readily used by the microbial community [1]. The nitrogen component of DOM (dissolved organic nitrogen, DON) is particularly important in marine biogeochemistry since nitrogen availability frequently limits marine primary production [2]. As a consequence, the capacity of bacteria to degrade DON and remineralise nitrogen (producing inorganic nitrogen forms) is crucial to support primary production where allochthonous nutrient supply is limited [3]. When accessing DOM, bacteria modify the structure of the DOM reservoir by preferentially using nitrogen over carbon [4, 5]. Consequently, the dynamics of DON use subtly differ from those of DOC use leading to changes in DOM stoichiometry as a consequence of bacterial activity. Given its relative carbon richness [C:N:P up to 3511:202:1; [4, 6]], most studies addressing DOM have focused on DOC [7] and much less is known about DON.

Phytoplankton are recognised as one of the main producers of DON in the marine environment through active exudation, passive release through cell membranes and release due to cell rupture (e.g. viral lysis [8]). However, marine DON production is also associated with heterotrophic processes such as grazing, particulate organic matter remineralization and bacterial release of extracellular enzymes [8]. In particular, heterotrophic bacteria are thought to mediate the production of slowly degradable DON (recalcitrant DON; RDON). McCarthy et al. [9] proposed that bacteria are the main source of RDON in the ocean through the release of peptidoglycan remnants derived from bacterial cell walls. More recently, Meon and Kirchman [10], investigating DOM production and stoichiometry, observed an accumulation of relatively N-rich dissolved combined amino acids (DCAA) by the end of their experimental mesocosm study. As DCAA increased when the mesocosm was shaded and no correlation with chlorophyll was observed, these authors concluded that the relatively recalcitrant DCAA were produced by heterotrophic degradation of organic matter following the growth of phytoplankton.

Despite these findings, the extent to which bacteria are able to utilize phytoplankton-derived DON was never specifically addressed. The scope of this work was to investigate the capacity of a widely distributed bacterium (*Alteromonas* sp.) to utilize phytoplankton-derived DON and DOC. To achieve this goal we combined laboratory experiments with modelling.

Using DOM derived from lysed cells of an axenic, exponentially growing culture of the marine diatom *Chaetoceros calcitrans*, we investigated how bacterial growth modified the concentration and stoichiometry of DOM. *Alteromonas* sp. was used for this study based on its ecological relevance and metabolic versatility [11]. We used an established model describing the interactions between DOM and bacteria [12] to test the hypothesis that part of the DON pool generated by phytoplankton was resistant to bacterial degradation over the investigated time scale.

## Materials and methods

Where appropriate, glassware and filtration/sterilisation equipment was cleaned in laboratory detergent, 10% HCL and then autoclaved prior to use. All chemicals were sourced from Sigma-Aldrich (UK) unless otherwise stated.

Phytoplankton cultures: The marine diatom *Chaetoceros calcitrans* fo. *pumilis* (CCAP 1010/11) was selected for the generation of a dissolved organic matter enriched media (DOM-EM). Stock cultures were maintained on artificial seawater (ASW) based media [13] using de-ionized and purified water (ELGA Purelab Ultra, VWS UK Ltd., High Wycombe, UK) for media preparation. Bicarbonate was added at 2.0 mmol∙L^-1^. Nitrate, phosphate and silicate were added at 100 μmol∙L^-1^, 5 μmol∙L^-1^ and 100 μmol∙L^-1^ respectively. No organic buffers were added. Enrichment solutions for vitamins, iron, and trace metals were added at 10% of the original formula [14] to decrease the amount of DOM added as ethylenediaminetetraacetic acid. Culture media was filter-sterilised by pumping it into autoclaved experimental flasks through sterile 47 mm diameter, 0.2 μm filters (Durapore; Merck Millipore, Billerica, MA, US). Stock *C*. *calcitrans* cultures were grown at 15°C, at a light intensity of 70 μmol∙s^-1^∙m^-2^ with a 16:8 light:dark (L:D) cycle. Axenicity of the stock cultures was checked regularly using analytical flow cytometry (see below) to ensure that *C*. *calcitrans* remained bacteria and virus-free.

To create DOM-EM, axenic *C*. *calcitrans* stock cultures were used to inoculate 20L ASW media volumes which were subsequently used to generate DOM-EM. Two 20L volumes of complete ASW were filter-sterilised (see above) into sterile borosilicate vessels (nominal volume of 24L). Media contained nitrate, phosphate and silicate at 100 μmol∙L^-1^, 5 μmol∙L^-1^ and 100 μmol∙L^-1^ respectively. DOC/TDN analysis was used to verify that the DOC/DON concentration was below detection (section 2.5). Screw-cap tops with 4-port valves capped the borosilicate vessels. Three of the four ports were used. One port allowed for the removal of sample volume. The second and third ports were attached via silicon tubing to air filtration units (Hepa-Vent, Whatman). Atmospheric air was pumped at a flow rate of 500 mL∙min^-1^ into culture vessels through the second port, entering the media via a Teflon tube within the culture vessel (no air-stone or equivalent was used; culture media was mixed by bubbled air). Air exited the culture vessel via the third port-valve (with disc filter attached, Hepa-Vent, Whatman). Sterilised complete media was placed in a constant temperature room at 15°C and illuminated at 70 μmol∙s^-1^∙m^-2^ with a 16:8 L:D cycle for 48 hours for thermal equilibration. Following thermal equilibration, a 2 mL sample was removed and tested using analytical flow cytometry (AFC; see below) to verify that the media was axenic. Media was then inoculated with *C*. *calcitrans* at an initial cell density of 7.6∙10^3^ cells∙mL^-1^.

Samples were collected from culture vessels on a daily basis for diatom cell density and inorganic nutrient concentrations. At late-exponential growth (approximately day 7) vessels were transferred to a freezing facility and cultures were left to freeze for 24 hours. Cultures were thawed, which typically took 48 hours. The freeze-thaw cycle was repeated twice.

Following freeze-thaw cycles, phytoplankton cellular material was removed by a series of filtration steps: 20L cultures were initially coarsely filtered (142 mm GF/C, Whatman), followed by sequential filtration through 47 mm GF/F units (Whatman) at a low flow rate (≈250ml∙min^-1^), followed by a final filtration step through 0.2 μm PES membrane filters (Supor®). Sterilisation of this media provided DOM-EM which was used for both the maintenance of bacterial stock cultures and experimental work. A 2 mL sample was removed and tested using analytical flow cytometry (AFC; see below) to verify that the DOM-EM was axenic.

Bacterial cultures: The marine bacterium *Alteromonas* sp. (originally isolated from station L4, Western Channel Observatory: WCO, www.westernchannelobservatory.org.uk), was obtained from the Plymouth Marine Laboratory microbial archive (Karen Tait, pers. comm.). An inoculum of *Alteromonas* sp. initially grown on marine broth (Difco medium 2216) agar plates, was established into liquid culture by adding to 1 L of sterile ASW-based growth broth containing 5% Bacto Peptone and 1% yeast extract. After 24 h, growth of *Alteromonas* sp. was confirmed (5.3∙10^7^ cells∙ml^-1^) using AFC analysis (see below), and 1 mL of this bacterial inoculum was then transferred into 500 mL of sterile DOM-EM (see above) to create a bacterial stock culture for the subsequent experiment. *Alteromonas* sp. stock cultures in DOM-EM were maintained at 15°C with a light intensity of 13 μmol s^-1^ m^-2^ and a 16:8 L:D cycle. Stock cultures were acclimated at these environmental conditions for at least 10 generations before inoculation into experimental vessels.

For experimental work, triplicate 5L borosilicate vessels with screw-cap 4-port valve tops, containing 5L volumes of sterile DOM-EM were used. Experimental vessels were placed in a CT room at 15°C for 48 hours to thermally equilibrate. At this point 2 mL samples were removed for bacterial analysis using AFC to test for contamination. Once cultures were verified to be axenic they were inoculated with 100 mL of *Alteromonas* sp. stock culture under sterile conditions.

### Experimental parameters

DOM-EM was inoculated with *Alteromonas* sp. at an initial cell density of 3.03∙10^5^ ± 3.0∙10^3^ (SD) mL^-1^. Experimental cultures were maintained at a constant temperature of 15°C and illuminated at 13 μmol∙s^-1^∙m^-2^ with a 16:8 L:D cycle. Culture development was monitored through periodic sampling by removing 350 mL for the assessment of bacterial abundance (AFC) and culture media chemical composition. From this 350 mL volume, a 2 mL sub-sample was used for AFC analysis and the remaining sample volume was filtered in a series of 100 mL volumes through pre-ashed 25mm GF/F filters. The filters were stored frozen and subsequently used for particulate organic carbon and nitrogen analysis.

The filtrate was pooled and a 2 mL sub-sample was retained for AFC analysis to derive the GF/F retention efficiency (required for mass spectrometry analysis; section 2.4). The filtrate was filtered through a 0.2 μm PTFE syringe filter to remove residual bacterial cells, and sub-divided between a series of sample volumes for further analysis. Approximately 75 mL of filtrate was retained in two 100 mL high density polyethylene bottles and stored frozen for subsequent inorganic nutrient analysis (section 2.3) and Dissolved Organic Carbon/Total Dissolved Nitrogen analysis (section 2.5). The remaining filtrate was used for analytical procedures which provided an indication of changes in DOM composition (Gas Chromatography Mass Spectrometry (GCMS), Liquid Chromatography Mass Spectrometry (LCMS); see supplementary information).

### Flow cytometric analysis

*Chaetoceros calcitrans* and *Alteromonas* sp. abundance were enumerated using a BD Accuri™ C6 flow cytometer following the methods of Tarran et al. [15] and Brussaard et al. [16]. Phytoplankton samples (1 mL) were analysed live without processing, immediately after sampling. For enumeration of bacteria, 1 mL samples were fixed with glutaraldehyde (0.5% final concentration) in 5 mL polypropylene tubes and then placed at 4°C for 30 minutes. Bacterial samples were then stained with SYBR Green I DNA stain (Life Technologies, Paisley, UK) following the protocol of Marie et al. [17] at a 1:10,000 final dilution of initial stock and potassium citrate (30 mM final concentration) dissolved in Milli-Q (Millipore, Billerica, USA) water, in the dark at room temperature for 1 h. For virus enumeration, fixed, frozen samples were defrosted at room temperature and then analysed using AFC following standard protocols [16].

Samples were analysed at a nominal flow rate of 100 μL∙min^-1^ and 30 μL∙min^-1^, for *C*. *calcitrans* and bacterial/virus populations respectively, and a core of 15 μm. The flow rate was calibrated on each sampling day using changes in volume [18]. Phytoplankton samples were analysed for 5 min and data acquisition was triggered on both chlorophyll fluorescence and forward light scatter. For analysis of Sybr Green I-stained bacteria and virus samples, data acquisition was triggered on green fluorescence and forward light scatter and samples were analysed for 1 min. Data analysis were made using CFlow Plus software (Becton Dickinson, Oxford) with log amplification on a seven-decade scale.

### Inorganic nutrient analysis

Dissolved Inorganic Nitrogen (DIN; nitrate, nitrite, ammonium), dissolved inorganic phosphate and silicate were measured colorimetrically with a 5-channel segmented flow Bran and Luebbe AAIII autoanalyzer, using methods described previously [19].

### Particulate Organic Carbon (POC) and Particulate Organic Nitrogen (PON) analysis

The concentration of particulate organic carbon and particulate organic nitrogen was assessed by continuous-flow mass spectrometry using a PDZ Europa Scientific ANCA-NT system coupled to a PDZ Europa Scientific 20–20 stable isotope analyser. Samples were calibrated against leucine.

### Dissolved Organic Carbon (DOC) and Total Dissolved Nitrogen (TDN) analysis

Dissolved organic carbon and nitrogen were analysed using a Shimadzu TOC-V system. Samples were exposed to high temperature catalytic combustion for quantitative oxidation. DOC was oxidised to CO_2_ gas and measured by a non-dispersive infrared gas analyser. Total dissolved nitrogen was oxidised to NO and reacted with O_3_ to give the radical NO_2_* species. Upon decay to the ground state the chemiluminescence was measured. Dissolved Organic Nitrogen (DON) concentration was derived by subtracting DIN concentration from TDN. A commercially available reference material was used for quality control (University of Miami, [20]).

### Model set-up

The model describing the interactions between DOM and bacteria [12] is described in S1 File. For the purposes of this work, the model was implemented in a zero dimensional framework to mimic the culture environment. To this end, the model was run only with bacteria and DOM for 100 days. Initial conditions (biomass, DOM and nutrients) were taken from experimental data. The model accounts for two fractions of recalcitrant DOM. The first is called semi-labile and is produced by phytoplankton and/or bacteria as release of excess of carbon. Model semi-labile DOM is used by bacteria within a time scale of ten days. The second fraction (the most recalcitrant) is the result of the release of bacterial capsular material and is assumed to cycle on a time scale of one hundred days. It should be noted that according to the more recent classification of recalcitrant DOM fractions [7] the two classes of model DOM should be both labelled as “semi-labile”. However, here we kept the original definition as given in Polimene et al. [12] to emphasise that the most recalcitrant fraction considered by the model (capsular material) is derived from bacteria only. Both semi-labile and capsular material DOM are assumed to be formed by carbon only. The model only accounts for labile DON. To test the hypothesis that a substantial fraction of the DON that originated from the phytoplankton culture was resistant to bacterial degradation, we initialised the model with 23 μmol∙L^-1^ of labile DON instead of 66 μmol∙L^-1^ observed at the beginning of the bacterial culture. The remaining 43 μmol∙L^-1^ of DON were assumed to be totally resistant to degradation within the time frame of the experiment and therefore not modelled. We also assumed that a fraction of DOC (284 μmol∙L^-1^over a total of approximately 1500 μmol∙L^-1^) was associated with this resistant DON, assuming a C:N ratio equal to the Redfield value of 6.6. The remaining DOC (1216 μmol∙L^-1^) was assumed to be semi-labile. Note that background values of refractory DOC and refractory DON were added to the simulated values for diagnostic purposes. Model parameters were taken from Polimene et al. [12]. However, to improve the agreement with the data, we manually tuned the model parameters describing the maximal bacterial growth efficiency (the actual efficiency is dynamically computed by the model) and the time scale of the release of semi-labile carbon (overflow metabolism). These two parameters were increased from 0.4 to 0.6 and from 1 to 10 days, respectively. Finally, as we tested that viruses were not present in the cultures, the parameter regulating the rate of viral-induced mortality (lysis) was set to zero.

## Results

**Fig 1:**
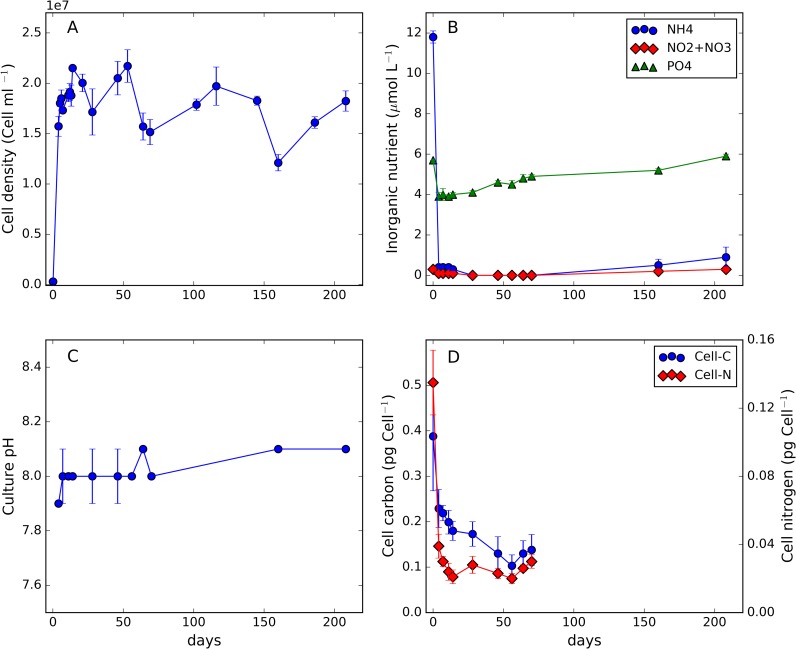
Temporal changes in cell density (A), inorganic nutrients (B), culture pH (C) and bacterial cell carbon and nitrogen content (D). Error bars indicate one standard deviation from the mean of triplicate measurements.

Changes in bacterial abundance for the 208 days of culture duration are presented in Fig 1, which includes nutrients, culture pH and measurements of bacterial cell carbon and nitrogen content. Bacterial cell abundance increased rapidly within the first few days of culture beyond which was observed a cycle of net increase and decrease in cell abundance with an extending period. Ammonium concentration decreased from ≈12 μmol L^-1^ to <0.4 μmol∙L^-1^ within 4 days of culture. Towards the end of the growth cycle (> day 160), NH_4_^+^ concentration increased from 0.15 μmol∙L^-1^ to 0.94 μmol∙L^-1^. Oxidised inorganic nitrogen remained <0.3 μmol∙L^-1^ for the duration of the experiment. Inorganic phosphate concentration decreased during the first few days of rapid bacterial growth (from 5.7 μmol∙L^-1^ to 3.8 μmol∙L^-1^) before increasing for the remainder of the experiment. Culture pH remained relatively stable at 8.04±0.07. Measurements of bacterial cell carbon and nitrogen content were only available for the first 70 days of the growth cycle. During this time, cell C-content decreased from 387±120 fg∙cell^-1^ to 122±6 fg∙cell^-1^. Cell nitrogen rapidly decreased from a value of 135±19 fg∙cell^-1^ to stabilise beyond day 11 at an average of 25±3 fg∙cell^-1^.

**Fig 2:**
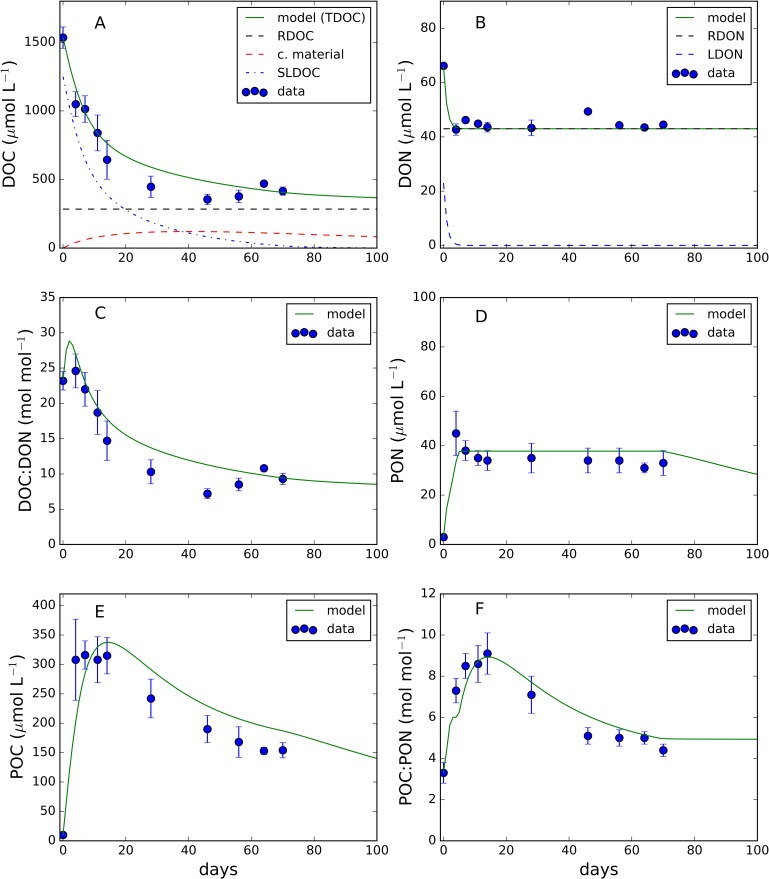
Observed and simulated temporal evolution of (A) total dissolved organic carbon (TDOC), (B) dissolved organic nitrogen (DON), (C) TDOC:DON ratio, (D) particulate organic nitrogen (PON), (E) particulate organic carbon (POC) and (F) POC:PON ratio. Simulations were carried out by using the model described in Polimene et al (12). Black dashed-lines in panel (A) and (B) indicate the fraction of DOC and DON assumed to be resistant to degradation by *Alteromonas* sp. (RDOC and RDON, respectively) within the time frame of the experiment. Blue dashed-line in panel (A) refers to the simulated semi-labile DOC (SLDOC). Red dashed line in panel (A) refers to the simulated DOC generated by the bacterial release of capsular material (c. material) (12). Blue dashed line in panel (B) refers to the labile DON (LDON) simulated by the model. See text for further explanation.

Fig 2 shows the observed and simulated values of DOM and carbon-nitrogen biomass variables from day 0 to day 70 when the full dataset (i.e. DOM and carbon and nitrogen biomass) was available. Additional measurements of DOC and DON were performed at days 160 and 208 and are discussed later in text. From an initial value of ≈ 1530 μmol∙L^-1^, DOC concentration (Fig 2A) rapidly decreased during the first 4 days, thereafter continuing to decrease at a lower rate until reaching a relatively stable value of ≈ 400 μmol∙L^-1^ by day 46. Afterwards, DOC concentrations remained approximately stable until day 70. The model (initialised as described above) was able to reproduce the observed trend. Simulated DOC concentrations, in particular, were very close to those observed at days 64 and 70 when they reached a value of approximately 400 μmol∙L^-1^. Fig 2A also demonstrated the simulated temporal evolution of semi-labile and bacterially produced capsular material. Model simulation suggested that the whole pool of semi-labile DOC was degraded by day 70 and that approximately 80 μmol∙L^-1^ of capsular material DOC were produced by bacteria in the same time frame. DON concentration (Fig 2B) decreased from a value of ≈ 66 μmol∙L^-1^ to ≈ 44 μmol∙L^-1^ within the first 4 days of growth, thereafter remaining stable for the duration of the experiment. The model captured the fast decrease of DON in the first days of the experiment. After day 4, simulated labile DON concentrations approached zero. The DOC to DON ratio (Fig 2C) for freshly produced phytoplankton DOM was ≈23; DOC:DON decreased to a value of ≈9 by day 46 where it remained relatively stable for the duration of the growth cycle. Simulated DOC:DON reproduced the general trend of the observations. However, the model overestimated the data between day 20 and day 60. The observed PON (Fig 2D) increased rapidly in the first 4 days while it remained roughly constant for the rest of the experiment; model simulation was in both qualitative and quantitative agreement with the measured data. Simulated and observed POC concentrations are presented in Fig 2E. Both observations and simulations show a steep increase in the first 15 days of the experiment. After day 20, observed POC decreased reaching a value of ≈150 μmol∙L^-1^ at day 70. Model simulations displayed a similar trend. However, POC was overestimated from day 20 onwards. POC to PON ratio (Fig 2F) increased from 4 up to 9 at day 17. After day 17 POC:PON decreased to a value of 4. Modelled POC to PON ratio reproduced the observations in terms of both quality and quantity.

DOC concentration at day 160 and 208 were 543±39 and 551±13 μmol∙L^-1^, respectively. DON concentration at day 160 and 208 were 48.7±1.9 and 51.7±1.6 μmol∙L^-1^, respectively. These values were higher than those measured at day 70 and indicated the lack of net bacterial degradation during the investigated timeframe.

## Discussion

Our results demonstrated that when provided with phytoplankton-derived DOM as the only energy source, *Alteromonas* sp utilised approximately 75% of the DOC. This was consistent with previous findings highlighting the versatility of this clade in accessing and degrading a wide range of DOC compounds [11]. However, approximately 65% of the initial DON was not used in the timeframe of the experiment. The DON pool concentration was relatively consistent after 4 days of growth suggesting that only a fraction of the initial DON was readily accessible and a residual concentration of approximately 44 μmol∙L^-1^ remained in the media. The decreasing DOC:DON ratio beyond the fourth day of growth demonstrated a preferential net removal of C-rich DOM in contrast to previous findings [4, 5]. The analysis of the cellular carbon to nitrogen ratio indicated that in the early stages of growth (days 0–20) bacteria were rich in carbon with C:N values higher than previously reported [21], although this C-sufficiency decreased as culture duration increased. Our interpretation of these results is that *Alteromonas* sp. lacked the metabolic machinery to readily degrade the residual diatom derived DON following the initial period of rapid utilisation and that this inability potentially constrained bacterial growth. By assuming that a component of DON was resistant to degradation (44 μmol∙L^-1^ of DON associated with 285 μmol∙L^-1^ of DOC, assuming Redfield stoichiometry) the model of Polimene et al. [12] reproduced the temporal evolution of the data (Fig 2) further supporting our interpretation.

In natural marine ecosystems phytoplankton is known to produce DON directly through active release or passive diffusion through cell membranes, and indirectly by means of trophic mediated processes (grazing) and viral lysis [8]. The DOM used in this study was not formed by algal exudates; instead, the method mimicked cell rupture induced by grazing or viral lysis. By adopting this approach our DOM-enriched medium reflected the complexity of the phytoplankton cell metabolome and would have been more chemically intricate than a media based only on DOM components actively or passively released by phytoplankton. The rationale for adopting this approach was to more rigorously test *Alteromonas*’ metabolic versatility. The ability of this species to use DOM generated in this way has not been (to the best of our knowledge) explicitly assessed to date and therefore there were no data to directly compare with our results. Nevertheless, following a period of rapid growth, this DOM sustained bacterial activity for > 6 months of culture (Fig 1A). The longer term gradual downward trend in cell abundance beyond day 7 was achieved via a series of net increases and decreases in cell abundance, possibly associated with cell death/nutrient release/nutrient regeneration cycles. During this phase, bacterial activity continued to modify the DOM pool composition as evidenced by changes in DOC/DON concentration and stoichiometry (Fig 2A–2C). Changes in nutritional quality and availability of residual DOM had implications for bacterial cell C:N (Fig 2F); bacterial cells were relatively N-limited/C-sufficient during the first 20 days. The rapid removal and utilisation of NH_4_^+^ (Fig 1B) and an accessible fraction of DON was insufficient to balance DOC uptake and utilisation during this phase. However, the regeneration of NH_4_^+^ observed (>160 days) and the relatively low values of particulate and dissolved C:N stoichiometry after day 40 suggested a shift toward carbon limitation during the later stages of the experiment. The regeneration of phosphate throughout the experiment following an initial net removal (Fig 1B) suggested that cells satisfied cellular P-demand by accessing DOP (not measured).

Although a persistent residual DON component (44 μmol∙L^-1^) was measured in our culture, this does not necessarily imply that this pool was stagnant, i.e. that it did not turnover. It is feasible that DON pool composition was modified by bacterial activity during the experiment despite the persistence of a net residual concentration. For example, it is possible that bacterial activity led to a transition in DON provenance from a phytoplankton to a bacterial origin without a significant change in residual concentration. Indeed, DON can be produced by bacteria through different processes. For example, the degradation of bacterial cells during grazing and/or viral lysis is thought to be a major contributor to the oceanic RDON pool via the release of peptidoglycan fragments [9]. However, in our study, the lack of both grazers and viruses made the contribution of the associated recycling processes to DON formation arguably low. Alternatively, the natural death of bacterial cells (i.e. autolysis, [22]) could have made a contribution to the production of DON which we are unable to quantify directly. Additional bacteria-mediated mechanisms of DON production reported in the literature are unlikely to contribute to DON formation in the early stages of our experiment; bacteria might release DON via active enzyme release and passive diffusion through the cell membrane [8]. However, this DON is likely to be labile (urea and amino acids) and is generally produced during growth deceleration [23, 24] whereas in our experiments DON stabilised during the exponential growth of bacteria. Bacteria are also known to release excess DOM generated by the uncoupling of catabolism from anabolism (overflow metabolism, [25]). However, the increase in POC:PON indicated that bacteria were limited by nitrogen between days 5–25 and this made the presence of excess DON within the cells unlikely. Finally, DON could have escaped from degradation because it was too dilute to be taken up by bacteria; recent studies [26] offered an explanation of apparent recalcitrance by means of the so called “dilution hypothesis” implying that DOM could be formed of labile molecules present at concentrations too low to compensate for the metabolic cost associated with their consumption. However, this mechanism was invoked to explain the recalcitrance of DOC in oceanic deep waters where organic matter concentration was lower than 55 μmol∙L^-1^ [26] and is unlikely to play a significant role in the system investigated here which is characterised by highly concentrated DOM.

For all these reasons, the most likely explanation for resilience of the observed DON was that *Alteromonas* sp lacked the metabolic versatility to fully accesses DON; having rapidly exhausted this capacity it was nevertheless able to continue accessing C-rich components of the DOM pool. It has been demonstrated that different bacterial groups may use carbon or nitrogen-rich compounds with different efficiencies. Jiao and Zheng [27] compared two of the major bacterial clades in the ocean, *Roseobacter* and SAR11 and showed that the former was more efficient in degrading DOC, while the latter preferred N-containing DOM. However, studies of this kind using *Alteromonas* species are, to the best of our knowledge, lacking.

An additional factor contributing to the lack of DON degradation may have been the decrease in lability of common N-containing compounds such as proteins and amino acids (free or combined) when bound to colloidal material. Nagata and Kirchman [28] observed that rates of protein utilization by bacteria strongly decreased when these compounds became associated with colloidal DOM (cDOM). Schuster et al. [29] estimated that up to 55% of the dissolved amino acids in marine waters could be associated with cDOM and that leucine uptake could be reduced by 2–3 orders of magnitude when bound to cDOM. In the marine environment cDOM production has been documented both from phytoplankton (during bloom decay [30, 31, 32]) and bacterioplankton [33, 34]. In our cultures, colloidal DOM could have been present in the original diatom-derived medium or could have been produced by bacteria via the release of capsular material [34]. While cDOC was not measured in our experiment, the Polimene et al. [12] model simulated the accumulation of bacterial capsular material during the experiment (Fig 2A). Drawing on the above cited literature and on model simulations, we hypothesise that the observed DON resilience to bacterial degradation reflected a combination of i) the chemical complexity of the phytoplankton metabolome which exceeded bacterial versatility and ii) the interaction between lysis-derived material and bacteria-derived DOM (capsular material). This would suggest that grazing and/or viral lysis of high productivity events [35] and the associated bacterial activity could produce DON resilient to further degradation due to the presence of colloidal DOC shielding labile N-containing molecules. However, the extent to which this result reflects the limitations of a single bacterial species rather than a bacterial community, as would naturally occur, requires clarification. Additional experiments explicitly assessing the presence of colloidal DOM and bacterial capsular material production are required to investigate this mechanism.

The modelling component of this work, other than contributing to the interpretation of the experimental results, provided a conceptual validation of the microbial component of a widely used marine ecosystem model (ERSEM, [36, 37, 38]). The model suggested that bacterial release of capsular material contributed to the recalcitrant DOM pool observed after 70 days (Fig 1A), consistent with the Microbial Carbon Pump concept [4]. In particular, the model simulated that approximately 8% of the initial semi-labile DOC was converted into more recalcitrant carbon by bacteria through the release of capsular material. While we could not quantitatively validate this specific model feature, the production of bacterially-derived DOM was at least conceptually confirmed by our analysis on the quality of DOM during the experiment using analytical techniques (S2 File.).

Two analytical approaches were used to investigate changes in DOM pool composition, both of which were limited to the first 70 days of bacterial growth; Gas Chromatography Mass Spectrometry (GCMS) and Liquid Chromatography High-Resolution Accurate Mass-Mass Spectrometry (LC/HRAM-MS). Offering a relatively narrow though consistent view of DOM pool composition, GCMS analysis demonstrated that the DOM pool was a dynamic reservoir modified by bacterial activity, with component(s) being rapidly or gradually removed, accumulated then removed, or left unmodified (Fig A in S2 File). Untargeted LC/HRAM-MS analysis demonstrated the complexity associated with DOM analysis and identified N-containing components derived from the diatom and/or the bacteria that were resilient to bacterial degradation.

Our results demonstrate the limitations of using a single bacterial cell line for the interpretation of DOM dynamics and are consistent with previous studies suggesting that a bacterial community is needed to operate in succession and synergy to degrade DOM in the marine environment [39]. Indeed, although *Alteromonas* species have been shown to have a highly versatile metabolism and to be able to consume as much labile DOC as a diverse free-living microbial community [11], an inability to efficiently degrade DON derived from lysed diatoms was demonstrated here. Additional experiments with different bacterial clades, grown under different environmental conditions are required to assess the extent of the impacts of these findings on the whole marine microbial community and to assess the potential recalcitrance of phytoplankton-derived DON.

## Supporting information

S1 Dataset Data Fig 1. (XLSX)

S2 Dataset Experimental data Fig 2. (XLSX)

S3 Dataset Model Simulation Fig 2. (XLSX)

S1 File Appendix 1. Model description.
(DOCX)

S2 File Appendix 2. DOM analyses.
(DOCX)
