# Supplementary material for: A substantial fraction of phytoplankton-derived DON is resistant to degradation by a metabolically versatile, widely distributed marine bacterium
Source: PLoS One. 2017 Feb 3;12(2):e0171391. doi: 10.1371/journal.pone.0171391 (PMC5291467; doi:10.1371/journal.pone.0171391)
Supplement: S1 File — Model description. (DOCX) [file pone.0171391.s004.docx]

**Model description**

Here we describe model equations and basic assumption which are more relevant for the purpose of this paper. We remand the reader to the published literature [1,2] for a complete description of the model.

The model describes the bacterial uptake of dissolved organic matter (DOM), the bacterial respiration, the bacterial production of recalcitrant DOM (RDOM) and the nutrient uptake/remineralization flux

*DOM uptake and respiration*

DOM is described through three distinct state variables: labile DOM (R1), semi-labile DOM (R2) and DOM derived from the release of capsular material (R3). R2 and R3 have higher turnover time with respect to R1 (i.e. they are recalcitrant DOM) and are assumed to be formed by carbon only. R1 (labile DOM) is assumed to be formed by C, N and P. Bacterial DOM-specific uptake is given by:

$B_{upt}=min({UPT}_{pot},r_{lab})$ (1)

Where ${UPT}_{pot}$ is the potential, substrate-specific uptake rate and $r_{lab}$ is the mass-specific turnover rate (d^-1^) of the labile fraction of DOM (R1), assumed to be 1 d^-1^. ${UPT}_{pot}$ is given by:

${UPT}_{pot}= \frac{{r_{bac}\cdot f}^{t}\cdot f^{O2}\cdot B_{c}}{R}$ (2)

Where $r_{bac}$ is the max bacterial growth rate, $B_{c}$ the bacterial carbon biomass and $f^{t}$ and $f^{O2}$ two non-dimensional functions accounting for the effect of temperature and O_2_ limitation, respectively [1]. $R$ is the total available carbon-substrate and is given by:

$R={R1}_{c}+ r_{sl}\cdot{R2}_{c}+ r_{sr}\cdot{R3}_{c}$ (3)

Where ${R1}_{c}$ ${R2}_{c}$ and ${R3}_{c}$ are the labile, semi-labile and capsular material component of the DOM, respectively. $r_{sl}$ and $r_{sr}$ are two factors which represent the turnover rate of ${R2}_{c}$ and ${R3}_{c}$ (respectively) relative to the turnover rate of ${R1}_{c}$

The absolute uptake of carbon is then given by:

$B_{upt}^{C}= B_{upt}\cdot R$ (4)

The absolute uptake of organic nitrogen is given by:

$B_{upt}^{N}= B_{upt}\cdot{R1}_{N}$ (5)

Where ${R1}_{N}$ is the nitrogen component of DOM (DON)

Bacterial respiration is assumed to be composed by two additive terms, the first ($B_{resp}^{A})$ describing the activity respiration and the second ($B_{resp}^{R})$accounting for the rest respiration:

$B_{resp}= B_{resp}^{A}+B_{resp}^{R}$ (6)

Where

$B_{resp}^{A}= B_{upt}^{C}\cdot\left( 1-Eff \right)$ (7)

Where $Eff$ is the fraction of carbon assimilated. $B_{resp}^{R}$ is given by:

$B_{resp}^{R}= r_{rest} \cdot f^{t}\cdot B_{c}$ (8)

Where $r_{rest}$ is the specific rest respiration rate.

*RDOM production*

RDOM is represented in the model trough the state variables R2 and R3. R2 is produced by bacteria in order to release excess of carbon when internal nutrients are in shortage and is given by:

${B_{R2}=max\left[ 0, max\left[ \left( 1-\frac{qpB}{P^{opt}} \right),\left( 1-\frac{qnB}{N^{opt}} \right) \right] \right]\cdot B}_{c}\cdot\mu_{rel}$ (9)

where *qpB* and *qnB* are the actual phosphorus (P) to carbon and nitrogen (N) to carbon ratios P:C and N:C intracellular ratio, respectively, and $P^{opt}$ and $N^{opt}$are the optimal P:C to carbon and nitrogen (N) to carbon ratios, respectively, and $\mu_{rel}$ (d^-1^) is the time scale of the processes

R3 production (i.e. DOM derived by the release of capsular material) is assumed to be proportional by a factor $f_{R3}$ to the activity respiration [3,1]:

$B_{R3}=f_{R3}\cdot B_{resp}^{A}$ (10)

*Nutrient dynamic (nitrogen)*

Bacterial nutrient dynamic is regulated by the values of the internal nutrient to carbon ratios *qnB* and $qpB$*,* relative to the optimal nutrient to carbon ratios ($N^{opt}, P^{opt}$). Hereafter only the nitrogen dynamic is described (phosphorous is treated in the same way):

${B^{nut}= \gamma\cdot\left( qnB-N^{opt} \right) \cdot B}_{c}\cdot f_{n}$ (11)

Where

$f_{n}=1$ if $qnB-N^{opt}>0$ (11.1)

$f_{n}= \frac{{NH}_{4}}{{NH}_{4}+ h_{n}}$ if $qnB-N^{opt}<0$ (11.2)

Where $h_{n}$ is the half saturation constant for ammonium uptake.

References

1. Polimene L, Zavatarelli M, Allen I. Model of interaction between dissolved organic carbon and bacteria in marine systems. Aquat Microb Ecol. 2006; 43:127-138

2. Butenschön M, Clark J, Aldridge JN, Allen JI et al. ERSEM 15.06: a generic model for marine biogeochemistry and the ecosystem dynamics of the lower trophic levels. Geosci Model Dev. 2016*;* 9: 1293–1339

3. Stoderegger K, Herndl GJ. Production and release of bacterial capsular material and its subsequent utilization by marine bacterioplankton. *Limnol Oceanogr. 1998;* 43(5):877–884
