# Supplementary material for: A substantial fraction of phytoplankton-derived DON is resistant to degradation by a metabolically versatile, widely distributed marine bacterium
Source: PLoS One. 2017 Feb 3;12(2):e0171391. doi: 10.1371/journal.pone.0171391 (PMC5291467; doi:10.1371/journal.pone.0171391)
Supplement: S2 File — DOM analyses. (DOCX) [file pone.0171391.s005.docx]

**DOM analyses**

**Growth dynamics for bacterial culture:**

Our experimental objective was to measure the influence of bacterial growth upon DOM pool concentration and composition. This precluded the use of seawater based media and constrained the choice of microbial cell lines to those amenable to growth on artificial seawater (ASW). The marine diatom *Chaetoceros calcitrans* and the marine bacterium *Alteromonas* spp. satisfied this requirement while also being ecologically relevant.

**Changes in DOM composition.**

Changes in DOM was investigated using two analytical approaches; Gas Chromatography Mass Spectrometry (GCMS) and Liquid Chromatography High-Resolution Accurate Mass-Mass Spectrometry (LC/HRAM-MS).

**GCMS analysis.**

GCMS analysis used methods developed from Lisec et al (2006). At each sampling point, 100 mL of filtrate was retained from each replicate, passed through a C18 solid phase extraction (SPE) cartridge (6 ml/500 g C18, Kinesis Ltd., UK) and stored frozen. In preparation for analysis, samples were thawed and eluted using methanol into 1.5 mL microcentrifuge tubes containing 50 µL of 0.1 mg·mL^-1^ C19:0 (Nonadecanoic acid). Tubes were centrifuged at 20,000G for 2 minutes. Supernatant was transferred to GC vials and blown to dryness under oxygen free nitrogen. Samples were stored for 24 hours over silica gel.

For GCMS analysis, samples were derivatized in 50 µL of MOA solution (50 mg methoxyamine in 5 mL pyridine) for 2 hours at 70°C. Subsequently, 2.5 µL of N-tert-butyldimethylsilyl-N-methyltrifluoroacetamide was added to samples and a second derivitisation at 70°C for 2 hours was undertaken. Samples were analysed using an Agilent Technologies 7890A Gas Chromatography unit coupled to an Agilent Technologies 5975C Inert XL Mass Selective Detector. Samples were resolved using an MDN column (30 m, 0.32 mm ID, 0.25µm film), a helium flow rate of 2.0 mL·min^-1^, and the following ramping profile; 80°C initial temperature ramped to 200 °C at 2 °C·min^-1^, ramped to 320 °C at 5 °C·min^-1^. Samples were ionised using electron impact and scanned in the *m/z* range 50-900.

Integrated peak area ratios were used to derive the concentration of resolved sample peaks using the internal standard reference. Assuming Redfield stoichiometry, a comparison with simultaneously measured DOC concentrations suggested that DOM retention efficiency varied in the range 4-18%. Consequently, and as would be expected, a range of SPE chemistries would be required to achieve a more comprehensive view. Results presented are therefore a relatively narrow, though consistent representation of changes in DOM pool components that were amenable to the analytical approach presented here.

Up to 150 peaks were typically identified in GCMS chromatographs (Fig S1A, B). Peaks were either individual compounds or groups of co-eluting compounds; this influenced the confidence (% probability) with which peaks could be identified by reference to fragmentation spectra libraries. Peaks in Fig S1 C-E are presented as area ratios to the internal standard.

A clear feature of the GCMS analysis was the rapid and selective removal of many peak compound(s) within the first 4 days of culture development (Fig. S1C). The peak compound(s) in Fig. 1C (P1-P3) had fragmentation spectra consistent with the fatty acids oleic acid (P1; probability 41%), stearic acid (P2; probability 38%) and hexadecanoic acid (P3; probability 85%). The forth peak (P4) could not be identified and most likely represented a complex mixture of compounds. Peak compound(s) P1 and P2 were removed rapidly by the bacterial cells while peak compound(s) P3 and P4 were used more gradually. Peak compound(s) in Fig S1D appeared not to be used by bacterial cells while peak compound(s) in Fig. S1E (P5, P6) appeared to accumulate up to T_28_ and then were gradually removed from the media.

These results demonstrated that the DOM pool was a dynamic reservoir. As the accessibility or nutritional value of DOM compounds was modified through bacterial activity, cells appeared to access other, potentially less energetically favourable compounds in order to maintain growth. This activity influenced DOM pool concentration, composition and stoichiometry.

**LC/HRAM-MS analysis.**

Marine seawater DOM is a complex mixture of organic components; many are unknown and not amenable to GC analyses. Therefore a preliminary untargeted investigation was carried out with Liquid Chromatography High-Resolution Accurate Mass-Mass Spectrometry (LC/HRAM-MS). In order to separate and detect as wide a range of component’s as possible, LC/HRAM-MS methods were investigated which enabled direct injection of the samples. No pre-analysis processing was undertaken (e.g. SPE); such procedures are often necessary to both concentrate samples and remove the high salt content as the latter impairs sample preparation and analysis. Sample loss associated with pre-analysis processing was thus avoided.

LC/HRAM-MS was carried out using a U3000 UHPLC liquid chromatography system, Q Exactive Focus mass spectrometer fitted with a heated electrospray ionisation source (HESI II) and Xcalibur 3.0.63 software for data acquisition and processing (Thermo Scientific, Hemel Hempstead, UK). The following LC/HRAM-MS conditions were used: Chromatographic separation was carried out using a Kinetex 2.6 µm EVO C18 100 Å column (100 x 2.1 mm, Phenomenex, Macclesfield, UK) at 50°C with a flow rate of 500 µL min^-1^. Injection volume was 100 µL with a needle wash of water/methanol (10:90 % v/v). Four gradient elution systems were used: 1) 0.1% formic acid in water to methanol (AqFA/MeOH); 2) 0.1% formic acid in water to acetonitrile (AqFA/ACN); 3) 0.025% ammonia in water to MeOH (AqAm/MeOH) and 4) 0.025% ammonia in water/ACN (AqAm/ACN, all solvents were LC/MS grade). The gradient elution profile was [time/%organic]: 0/0
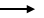
6/100
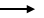
8/100
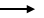
8.1/0
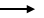
10.1/0 with the flow set to waste for the first 1.5 min. Due to the gradient delay volume the first 2 minutes (approximately) was an isocratic elution of 100% aqueous phase. Consequently, the four chromatographic gradient elution’s comprised of both acidic and basic 100% aqueous (Aq) to 100% methanol or acetonitrile organic eluents.

The mass spectrometer settings were: sheath gas (53 Arb, nitrogen), auxiliary gas (14 Arb, nitrogen), sweep gas (3 Arb, nitrogen), vaporiser temperature (300 °C), polarity (positive or negative ion), spray voltage (+3500/ -2500 V), capillary temperature (270°C) and S-lens RF level (50). The resolution was set at 70,000 @ *m/z* 200 with a full scan (*m/z* 100 -1000), AGC target (1e6, automatic gain control) and micro scans (1). External mass calibration (≤3 ppm) was used; auto-calibration using Pierce LTQ Velos ESI positive ion calibration solution (n-butylamine, caffeine, MRFA, and Ultramark 1621 (Thermo Fisher Scientific, UK)) and Pierce LTQ Velos ESI negative ion calibration solution (sodium dodecylsulfate, sodium taurocholate and ultramark 1621 (Thermo Fisher Scientific, UK)).

LC/HRAM-MS analyses of the ASW (i.e. artificial media complete with inorganic nutrients, vitamins and trace metals) and T_0_ -T_70_ samples were carried out using the four gradient elutions with positive ion full scan [*m/z* 100 -1000] detection. The data obtained were highly complex and will be the subject of further studies for targeted and untargeted interpretation and method development, which will include MS/MS for higher confidence structural identification and targeted compounds for quantitative and/or semi-quantitative analysis. Here we show preliminary (and tentative) results to demonstrate the applicability of the LC/HRAM-MS technique for the separation and detection for a wide range of compounds using an untargeted approach.

State-of-the-art chromatographic phases such as the Kinetex C18 EVO enabled the use of 100% aqueous and high pH basic eluents such as ammonia (≈ pH10 for 0.025% ammonia). This enabled the chromatographic retention of compounds not normally retained with even very low organic solvent in the mobile phase, or with acidic conditions. High pH can also be beneficial for the production of gas phase ions with both positive and negative electrospray ionisation achieving an increase in MS sensitivity. An advantage of being able to start with 100% aqueous eluents is the ability to retain highly polar compounds whilst un-retained salts can be prevented from entering the ionisation source using a divert valve to bypass the solvent front time period, thus preventing detrimental ionisation issues or MS contamination. This allowed direct injection of high inorganic salt samples such as marine waters without pre-treatment and with larger injection volumes for increased sensitivity. Here we compare four peaks of interest.

Comparison of the T_0_ base peak chromatograms (BPC) for the four elution methods (Figure S2) showed significant differences in observable peaks (although for all methods many of the components of interest do not show as peaks above the baseline due to the relative abundance of background ions). A high abundance peak was observed at 2.36 min in the acidic AqFA/MeOH gradient BPC (‘Peak 1’, Figure S2A) which corresponded to an *m/z* 130.15916 ion (Data not shown). With a HRAM ≤3 ppm, Xcalibur software calculated a molecular formula of C_8_H_20_N (with a ring double bond (RDB) equivalent of -0.5 and a Δ 0.134 ppm). As no other adducts were observable, this compound could have been either due to a quaternary ammonium M^+^ or protonated amine [M + H]^+^ compound with no double bond or ring moieties. Extracted ion chromatograms (EIC, *m/z* 130.15916, data not shown) showed that the peak at 2.37 min (AqFA/ACN, Figure S2B), 5.69 min (AqAm/MeOH, Figure S2C) and 5.32 min (AqAm/ACN, Figure S2C) was due to the same C_8_H_20_N compound. With acidic conditions in both MeOH and ACN, the compound eluted in virtually 100% aqueous acid eluent (gradient hold up time is approximately 2 min) whilst under basic conditions the compound was retained and eluted with both high MeOH and ACN compositions. The relative abundance of this ion (*m/z* 130.15916) did not appear to increase or decrease over the experimental time period (T_0-70_; T_70_ BPCs shown in Figure S3), suggesting that this diatom-derived N-containing compound was resistant to bacterial degradation.

A broad and high abundance peak at R_t_ 2.01, was retained and eluted during the 100% aqueous phase of the basic AqAm/ACN gradient T_0_ and T_70_ BPCs (Peak 2, Figures S4C and S5C). The observed ion for the peak was *m/z* 167.09277 (M^+^ or [M + H]^+^, C_7_H_11_ON_4_, RDB 4.5 and Δ 0.1894 ppm). The ion was not observed in the two acidic elutions (Figures S2A-B and S2A-B) and was either not retained or eluted during the first 1.5 min in which the eluent was diverted from the MS. In the basic AqAm/ACN gradient (Figures S2D and S3D) the ion was retained, but eluted whist the divert valve switched back to the MS. The relative abundance of this compound, which had a high molar nitrogen content (C:N =7:4) appeared to remain constant throughout the experimental time frame. As noted for ‘Peak 1’, ‘Peak 2’ may also represent a N-containing diatom-derived compound which was resistant to bacterial degradation.

The highest peak (R_t_ 5.17 min) in the AqFA/MeOH gradient T_0_ BPC (‘Peak 3’, Figure S2A) was due to ions *m/z* 214.08965 ([M + H]^+^, C_10_H_16_O_2_NS, RDB 3.5 and Δ 0.112 ppm) and *m/z* 236.07150 ([M + Na]^+^, C_10_H_15_O_2_NSNa, RDB 3.5 and Δ -0.782 ppm, data not shown) indicating a compound [M] with the formula C_10_H_15_O_2_NS (which could be n-butyl benzenesulfonamide; a common plasticiser, possibly derived from 0.2µm membrane filters). The *m/z* 214.08965 and *m/z* 236.07150 ions were also observed in the three other gradients at R_t_ 4.68, 5.18 and 4.66 min (‘Peak 3’, Figures S2B-D and S3 B-D respectively). The relative abundance of this compound appeared to remain constant throughout the experimental time frame. Curiously, this peak was not detected in the ASW blank (i.e. the medium used for diatom growth) although media and samples were treated in an identical manner. Consequently, the possibility that this compound was also derived from the diatom or the bacterium (i.e. was not a plasticiser) and was resistant to bacterial degradation remains open.

An obvious increase in relative peak height (5.50 min) was observed between the AqFA/MeOH gradient T_0_ and T_70_ BPCs (‘Peak 4’, Figures S2A and S3A). The corresponding mass spectra (data not shown), *m/z* 223.09666 ([M + H]^+^, C_12_H_15_O_4_, RDB 5.5 and Δ 0.782 ppm) and *m/z* 245.078367 ([M + Na]^+^, C_12_H_14_O_4_Na, RDB 5.5 and Δ -0.245 ppm) indicated a compound [M] with the formula C_12_H_14_O_4_. This would be consistent with diethylphthalate or similar phthalate and may have increased due to degradation of longer chain phthalates which are common contaminants from plastics. The increase was also observed in the three other gradients at R_t_ 4.91, 5.51 and 4.90 min (‘Peak 4’, Figures S2B-D and S3 B-D respectively). These results suggest that bacterial (or possibly diatom) derived enzymes were capable of degrading plasticiser contaminants.

The comparisons drawn here demonstrated the versatility of the methods; small highly polar nitrogen containing compounds could be separated and detected. Such compounds would not generally be retained with conventional C18 phases. The methods further demonstrated the influence that different mobiles phases had upon the ability to resolve sample components.

**References**

Pedler, B.E., Aluwihareb, L.I., Azama, F. 2014. Single bacterial strain capable of significant contribution to carbon cycling in the surface ocean. *PNAS*. 111:7202-7207.

Lisec, J., Schauer, N., Kopka, J., Willmitzer, L., Fernie, A.R. 2006. Gas chromatography mass spectrometry–based metabolite profiling in plants. *Nature protocols.* 1:387-396.

**Figures**


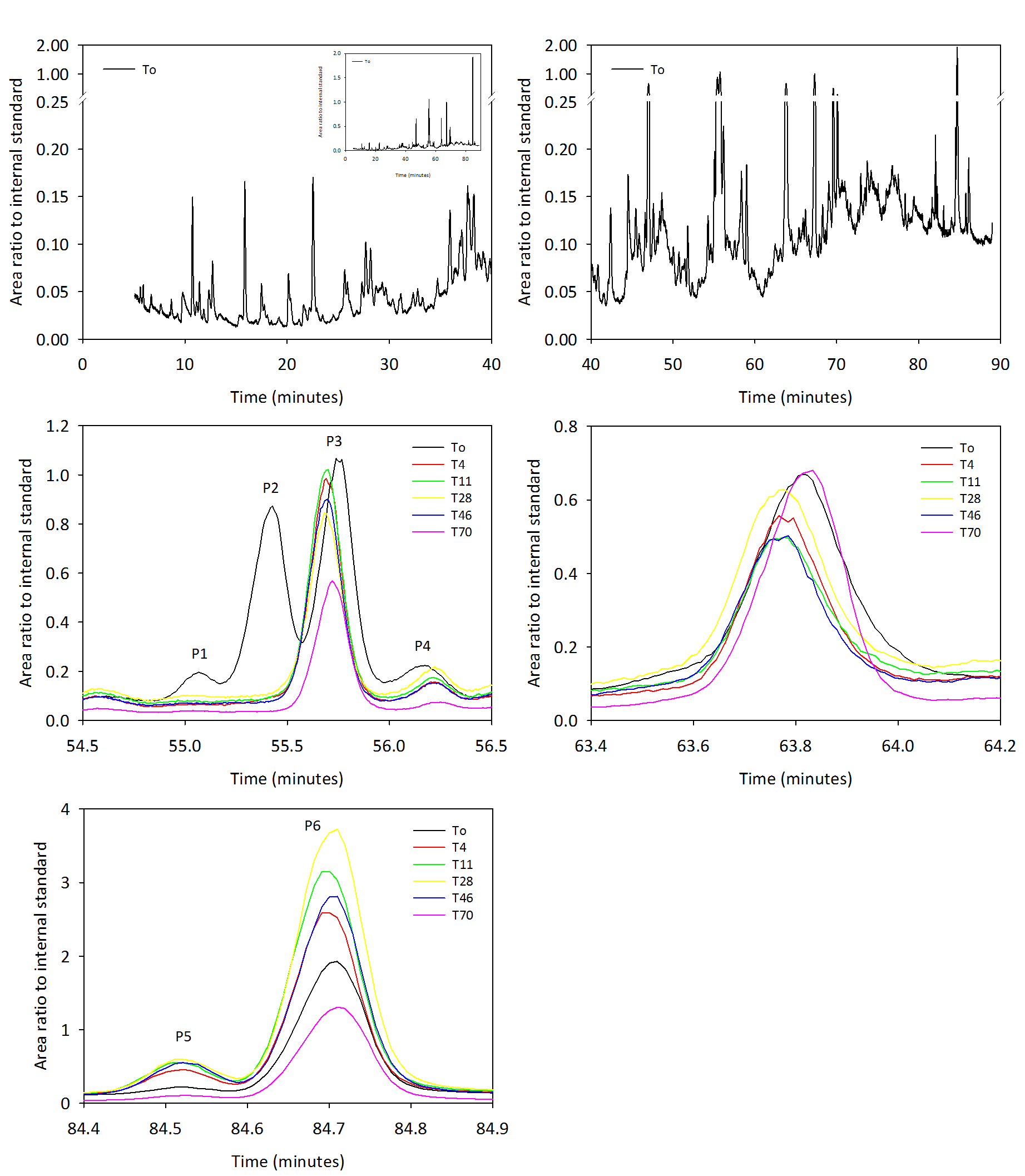


Fig. A. GCMS plots. A full chromatograph of T_0_ is split over 2 panels to facilitate viewable detail (A, B, the insert on panel A is the complete T_0_ to give an indication of scale). The removal of peak compound(s) over different time frames is presented in panel C. The persistence of peak compound(s) is presented in panel D. The accumulation and subsequent removal of peak compound(s) is presented in panel E.


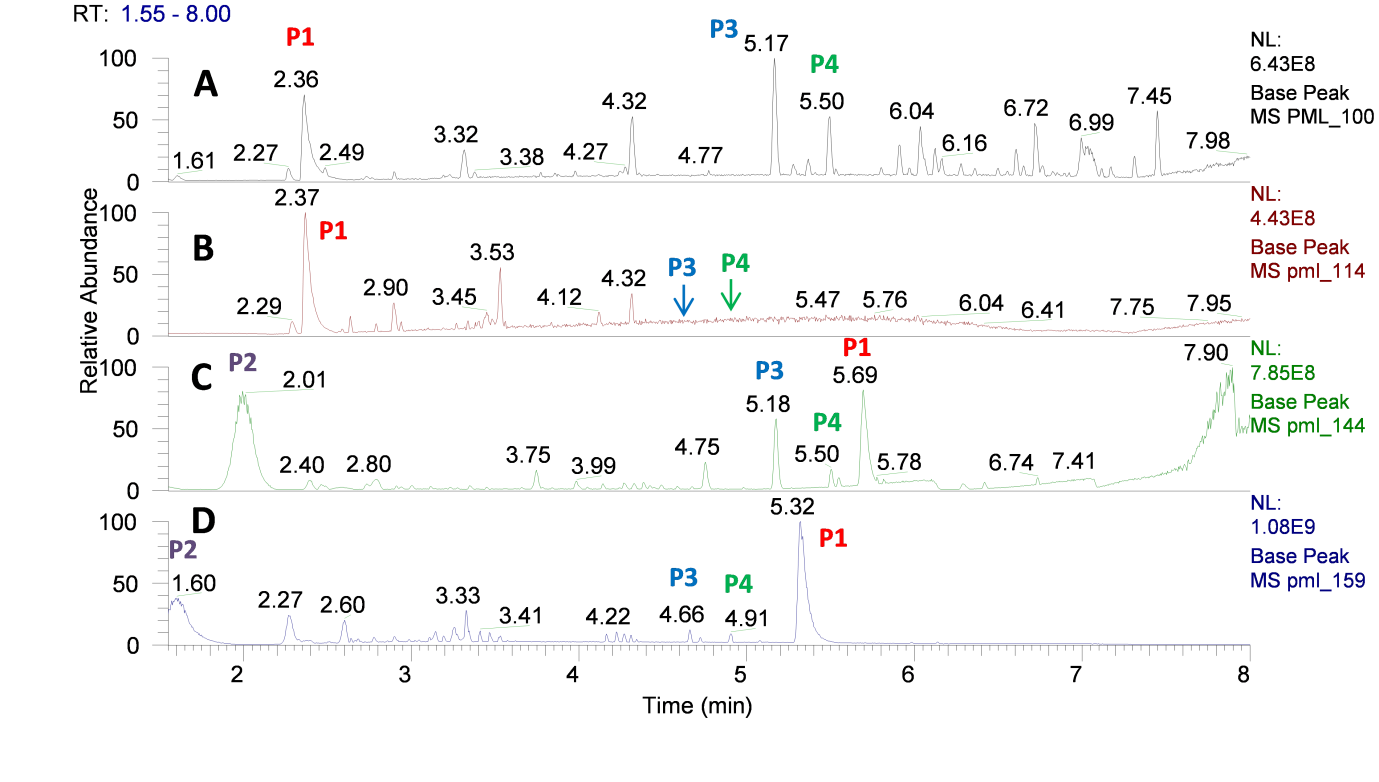


Fig. B. T_0_ LC/HRAM-MS gradient elution (100% Aq to 100% organic, Positive ion full scan [*m/z* 100 -1000] ) base peak chromatograms: A) AqFA to MeOH; B) AqFA to ACN; C) AqAm to MeOH and D) AqAm to ACN.


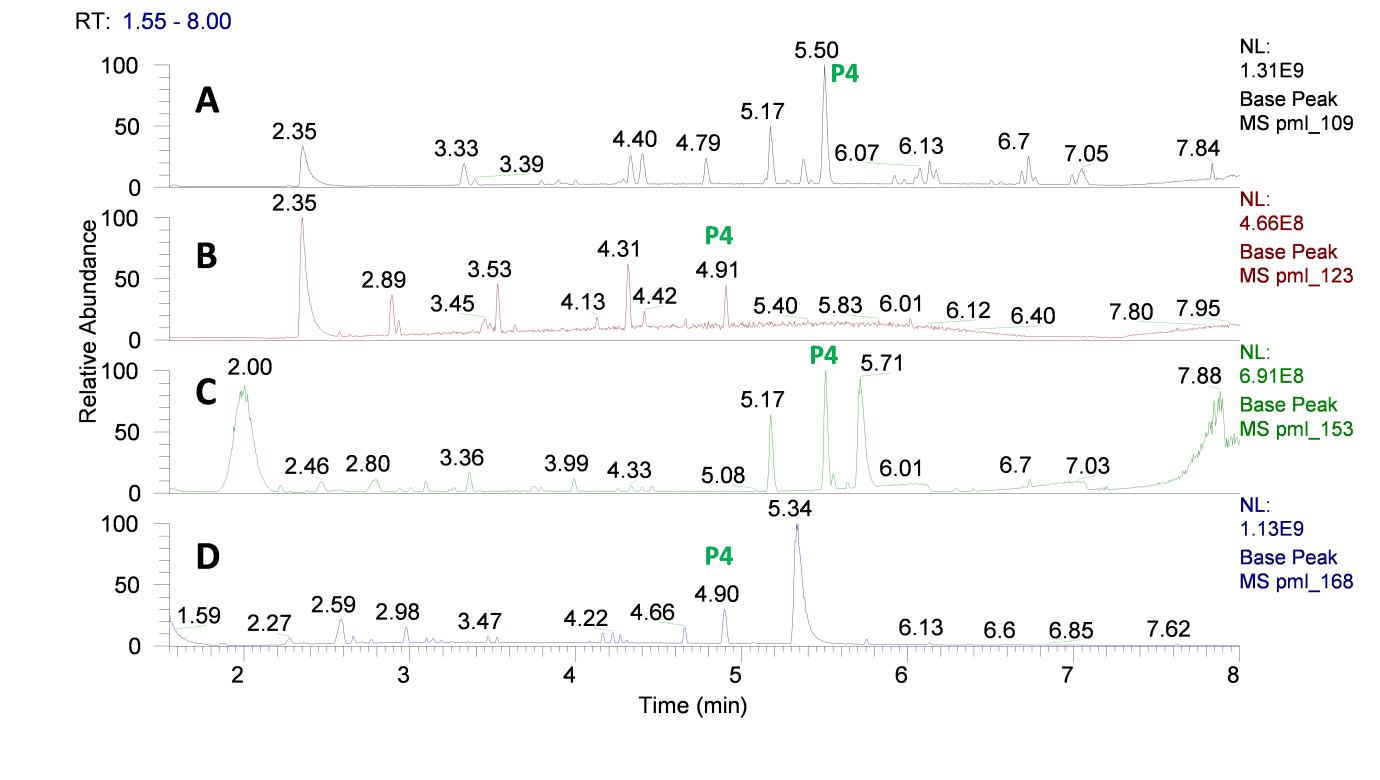


Fig. C. T_70_ LC/HRAM-MS gradient elution (100% Aq to 100% organic, Positive ion full scan [*m/z* 100 -1000] ) base peak chromatograms: A) AqFA to MeOH; B) AqFA to ACN; C) AqAm to MeOH and D) AqAm to ACN.
